# Exometabolite niche partitioning among sympatric soil bacteria

**DOI:** 10.1038/ncomms9289

**Published:** 2015-09-22

**Authors:** Richard Baran, Eoin L. Brodie, Jazmine Mayberry-Lewis, Eric Hummel, Ulisses Nunes Da Rocha, Romy Chakraborty, Benjamin P. Bowen, Ulas Karaoz, Hinsby Cadillo-Quiroz, Ferran Garcia-Pichel, Trent R. Northen

**Affiliations:** 1 Environmental Genomics and Systems Biology Division, Lawrence Berkeley National Laboratory, 1 Cyclotron Rd, Berkeley, California 94720, USA; 2 Climate and Ecosystems Sciences Division, Lawrence Berkeley National Laboratory, 1 Cyclotron Rd, Berkeley, California 94720, USA; 3 Department of Environmental Science, Policy and Management, University of California Berkeley, Berkeley, California 94720, USA; 4 School of Life Sciences, Arizona State University, 427 E Tyler Mall, Tempe, Arizona 85287, USA; 5 Molecular Cell Physiology Department, Faculty of Earth and Life Sciences, VU Amsterdam, de Boelelaan 1085, 1081HV Amsterdam, The Netherlands; 6 DOE Joint Genome Institute, 2800 Mitchell Dr., Walnut Creek, California 94598, USA

## Abstract

Soils are arguably the most microbially diverse ecosystems. Physicochemical properties have been associated with the maintenance of this diversity. Yet, the role of microbial substrate specialization is largely unexplored since substrate utilization studies have focused on simple substrates, not the complex mixtures representative of the soil environment. Here we examine the exometabolite composition of desert biological soil crusts (biocrusts) and the substrate preferences of seven biocrust isolates. The biocrust's main primary producer releases a diverse array of metabolites, and isolates of physically associated taxa use unique subsets of the complex metabolite pool. Individual isolates use only 13−26% of available metabolites, with only 2 out of 470 used by all and 40% not used by any. An extension of this approach to a mesophilic soil environment also reveals high levels of microbial substrate specialization. These results suggest that exometabolite niche partitioning may be an important factor in the maintenance of microbial diversity.

## Abstract (web summary)

Production and consumption of metabolites by soil microorganisms are important for nutrient cycling and maintenance of microbial diversity. Here, Baran *et al*. study metabolite uptake and release by desert soil microorganisms, showing that coexisting microbes can have divergent substrate preferences.

## Graphical abstract

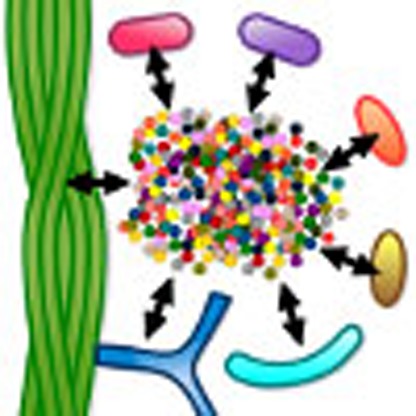

Environmental genomic surveys have demonstrated tremendous diversity among soil bacteria, and numerous factors contributing to the maintenance of this diversity have been proposed, including dormancy^1^, spatial isolation due to fragmentation of the soil aqueous phase^2^, and resource availability^3^ or partitioning^4^. A better understanding of the factors that underlie patterns of microbial diversity will improve our ability to predict the soil-catalysed biogeochemical cycles and manage agricultural practices accordingly. Soil microbial composition, with thousands of diverse bacterial taxa occurring in a single gram^5^,^6^, is dynamic and is known to vary with environmental factors and anthropogenic disturbance^7^,^8^,^9^. To date the relationship between microbial diversity, the composition and transformation of soil organic carbon and the impact of environmental change remains enigmatic.

Traditionally, soil carbon was thought to be composed of complex humics produced by *in situ* polymerization of plant and microbial metabolites. Nanoscale imaging and isotopic studies have led to the emerging view that old soil carbon is largely composed of complex pools of microbial metabolites associated with varying affinity to mineral surfaces^10^,^11^. According to the competitive exclusion principle, ‘complete competitors cannot coexist'^12^,^13^ and from this it has been suggested that in a homogeneous environment the maintenance of microbial diversity depends on the number of resources that exert dynamical effects on the populations^14^. While soils are certainly not homogeneous, resource competition can become an important factor under saturated water conditions that result in high connectivity^15^. Considering this, the production and consumption of diverse exogenous metabolites by soil microorganisms is likely important both for understanding the maintenance of microbial diversity and the turnover of soil organic matter.

Examining growth on single substrates and simple substrate mixtures has been an active area of research for over 100 years^16^,^17^,^18^, and supports the view of exometabolite niches. Related analysis of substrate utilization patterns (for example, Biolog)^19^ based on growth or respiration of microbes grown on isolated substrates has been widely used for characterization of isolates and microbial communities^20^. For example, data analysis from a study of ∼200 subsurface isolate strains grown on the 95 substrate Biolog GN plates^21^ found that the median number of substrates used by the strains was 18/95 (20%; ref. 22). Related studies on microbial growth have suggested that isolates on copiotrophic (rich) media have narrower substrate preferences than those obtained on oligotrophic media^22^ and that growth depends both on the specific metabolites (resources) present in their environment and on their concentrations^23^,^24^.

Unlike these early studies that have focused on single substrate utilization, technological advances in exometabolomics^25^ (metabolic footprinting^26^) now enable characterization of microbial metabolite utilization from mixtures of hundreds of metabolites with relevant composition and concentrations for a particular environment of interest. Exometabolomics has the potential to delineate microbial exometabolite niches^25^,^27^ for co-occurring (sympatric) bacteria based on observed specialized and preferential use of specific metabolites from the exometabolite pool (Fig. 1), helping couple soil metabolite composition to microbial diversity, and improve our understanding of soil trophic webs and nutrient cycling^28^. This interrogation of the extent of microbial utilization from relevant substrate mixtures addresses limitations associated with the single substrate utilization patterns^22^ and enables the examination of the utilization of novel or unexpected metabolites—such as the utilization of ergothioneine by *Shewanella oneidensis*^29^. This may also be relevant to uncultured microorganisms, where dependence on novel metabolites and multiple substrates may be the rule not the exception^30^,^31^ particularly under low nutrient conditions^32^.

Biological soil crusts (biocrusts) are microorganism-dominated communities in the top strata of soil that develop in areas where plant growth is restricted, notably in arid environments^33^,^34^; they provide a powerful system for exometabolomic investigation of transformations of microbial metabolites. The system is relatively simple since the primary producers and ultimate source of dissolved carbon are microbes. These communities persist in a desiccated, dormant state for extended periods of time and experience pulsed periods of activity following infrequent rainfall often saturating soils^35^. This enables collection of largely intact and dormant communities for laboratory experimentation. It is well known that the trophic web within early successional crusts is centred around the filamentous cyanobacterium *Microcoleus vaginatus*, which is considered to be the key primary producer in these biocrusts from the Colorado Plateau^36^. Since there is no evidence of atmospheric nitrogen fixation by *M. vaginatus*^37^, metabolic interactions with nitrogen fixers and other bacteria must be key for long-term viability of these biocrusts in these nitrogen-limited arid environments. As is the case in most soil communities, it is unknown if, in addition to neutral ecological processes like dispersal or genetic drift^38^, available carbon source composition and metabolic interactions may structure the microbial composition through processes such as competitive exclusion or niche differentiation.

Here we present an exometabolomic study of seven sympatric bacterial isolates from desert biocrusts aimed at characterizing their substrate preferences and potential metabolic interactions to test if metabolite diversity may support microbial diversity. Along with *M. vaginatus* (strain PCC 9802) originally isolated from early stage crusts in the Colorado Plateau^39^, we isolated six heterotrophic bacteria spanning three phyla that are common biocrust heterotrophs, phylotypes of taxa physically associated with the filamentous cyanobacteria and all isolated off the same media^40^. Since a large fraction of soil organic carbon is thought to be of microbial origin^10^, metabolites from the lysed cell metabolite extracts of the seven selected isolates were used as media supplements in exometabolomic experiments to simulate metabolites expected to be present in the natural environment including many yet unknown compounds. Metabolites from cell extracts of isolates were selected as supplements (in favour of metabolites from spent media) as these are easier to concentrate (centrifugation) and extract without simultaneous concentration of salts present in the media. The inferred metabolic transformations were then compared with those detectable during a laboratory biocrust activity pulse event to compare the metabolic capabilities of the isolates versus those in the intact community. While the idea that microorganisms have divergent substrate preferences is not new, here we provide what is to our knowledge the first experimental analysis of metabolite uptake and release of a broad range of metabolites present in their environment.

## Results and Discussion

### *M. vaginatus* releases a broad range of metabolites

**Figure 2:**
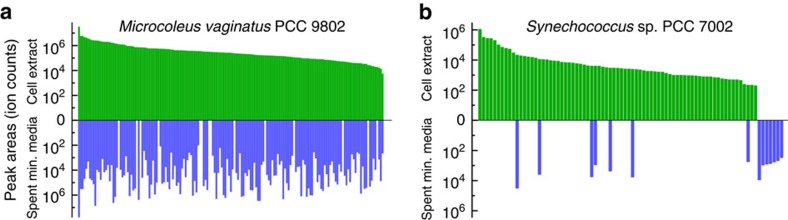
*M. vaginatus* PCC 9802 releases a broad range of metabolites. Comparison of metabolite levels in cell extracts and spent minimal media extracts of *M. vaginatus* PCC 9802 (**a**) and *Synechococcus* sp. PCC 7002 (**b**). Bars in each mirror plot represent ion counts of individual metabolites in cell extracts (green) or spent minimal media (blue). Metabolites are sorted in descending rank-abundance order according to ion counts in cell extracts. Data from a previous study^41^ were used for panel (**b**).

To assess metabolite release by the primary producer, *M. vaginatus* was cultured in minimal BG-11 medium and the cell extract, as well as spent medium were analysed by liquid chromatography—mass spectrometry (LC−MS). A broad range of metabolites was detected in both cell extracts and spent media, with only a small number of metabolites detected exclusively in the cell extract (Fig. 2a). Photoautotrophs are known to release metabolites^41^,^42^ though the breadth of metabolites released by the *M. vaginatus* greatly exceeds what we observed in our previous study of a unicellular euryhaline cyanobacterium *Synechococcus* sp. PCC 7002 (Fig. 2b)^41^. The metabolites detected in *M. vaginatus* cell extract but not (or detected at negligible levels) in the corresponding spent media include citrulline, mercaptohistidine betaine and a dihexose (Fig. 2a). This suggests that this metabolite release is not due to cell lysis as all intracellular metabolites would be expected in the spent media as well. While we cannot discriminate between leakage versus specific transport, ecologically, *Microcoleus* occupies a structured (soil) environment and may benefit from cross-feeding sympatric microbes versus the euryhaline *Synechococcus* sp.

Regardless of the mechanism, the harsh biocrust environment that includes rapid wetting and freezing would be expected to cause leakage and lysis. Given the abundance of *M. vaginatus* in the community^36^ these metabolites would represent a significant and richly varied resource for its neighbouring bacterial heterotrophs analogous to that seen in root zone (rhizosphere). Stimulation of such heterotrophs may result in the recruitment of bacteria with complementary functions (for example, heterotrophic nitrogen fixers) and may represent a strategy of *M. vaginatus* to outcompete diazotrophic cyanobacteria known to colonize later successional stages of biocrusts^43^,^44^.

### Crust isolates use subsets of metabolites

**Figure 3:**
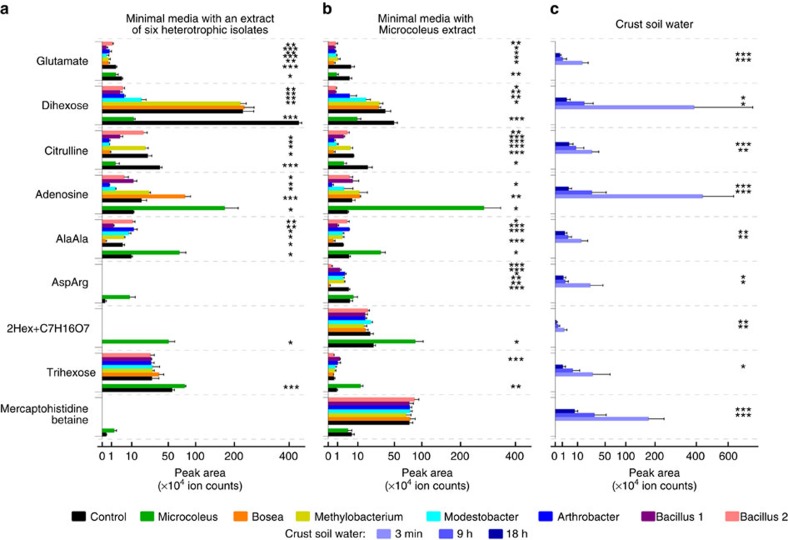
Uptake and release of selected metabolites by biocrust isolates and authentic biocrust. (**a**,**b**) Individual isolates were cultured in minimal media supplemented with a pooled metabolite extract of six heterotrophic isolates (**a**, Table 1) or metabolite extract of *M. vaginatus* PCC 9802 (**b**). Levels of metabolites in spent media (coloured bars) were compared with control media (no cells, black bars) to detect uptake or release of corresponding metabolites (*n*=3). A separate set of control media (black bars next to green bars) were used for *Microcoleus* spent media as *Microcoleus* was cultured for a significantly longer time than the heterotrophs. (**c**) Levels of selected metabolites in crust soil water extracts (Supplementary Fig. 3) are also shown (*n*=8). Error bars correspond to standard deviations. Stars correspond to Student's *t*-tests between spent media (coloured bars) and the corresponding control media (black bars; *P<0.05; **P<0.01; ***P<0.001). Supplementary Fig. 2 shows these comparisons for additional metabolites. The symbol 2Hex+C7H16O7 corresponds to a condensation product of a dihexose with a C_7_H_16_O_7_ residue.

**Figure 4:**
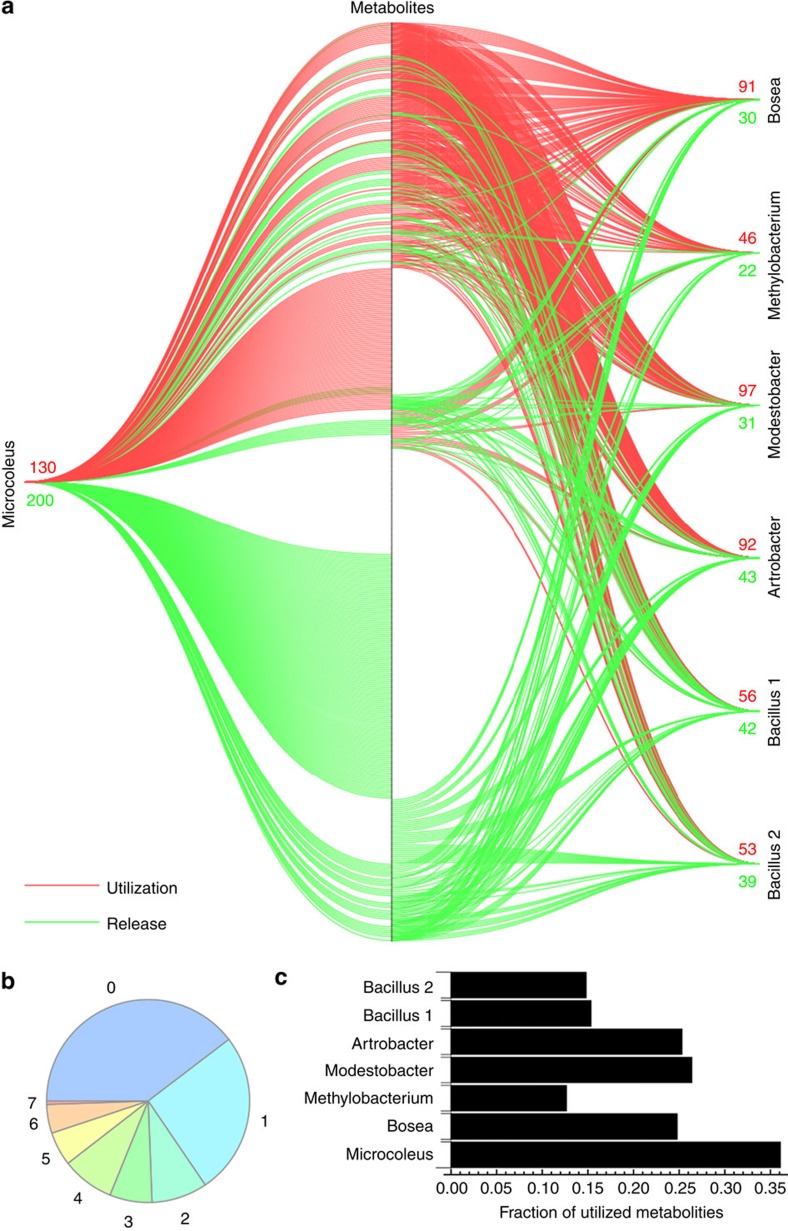
Selected biocrust isolates use only subsets of metabolites. (**a**) Lines between individual isolates and 470 metabolites detected in exometabolomic experiments in supplemented media represent utilization (red) or release (green) of a specific metabolite by given isolate. (**b**) Numbers of isolates taking up corresponding fractions of observed metabolites. (**c**) Fractions of metabolites taken up by individual isolates.

We then selected seven isolates obtained from these biocrusts for study (Table 1) to include diverse and common phyla (Actinobacteria, Cyanobacteria, Firmicutes and Proteobacteria) for examination of their substrate preferences. While we cannot specify the significance of these individual isolates to the community, sequencing of the ‘cyanosphere' community attached to the cyanobacterial sheaths shows that phylotypes of these isolates are in close physical association with the primary producers (Supplementary Fig. 1). The isolates were cultured individually in two types of complex medium to assess the scope of metabolite utilization. Here BG-11 minimal media was supplemented with either pooled cell extracts of the six heterotrophic isolates or with a cell extract of *M. vaginatus*. The exometabolomes from spent media were then profiled using LC−MS. Controls were run for abiotic transformation on uninoculated flasks of the corresponding supplemented media. Analysis of the raw data led to the annotation of 470 putative distinct metabolites (non-redundant ions of chromatographically separated components) across all media formulations (Supplementary Data 1). As is common in untargeted metabolomics, a large fraction of these metabolites could not be assigned, many of which are likely 'novel' compounds. Seventy-nine metabolites were identified based on our previous studies^25^,^41^,^45^ and MS/MS data (Supplementary Data 1). Chemical formulas were assigned to an additional 62 metabolites (Supplementary Data 1). Peak areas of characteristic ions of these metabolites in spent media were compared with those in the corresponding control media to detect utilization or release of these metabolites by specific isolates (Figs 3a,b,4a and Supplementary Fig. 2, Supplementary Data 1).

To evaluate metabolite utilization, including the utilization of 'novel' compounds, we focused on 372 abundant metabolites (the peak areas of their characteristic ions of at least 5,000 counts in any one of the control media). Overall, we find a high degree of metabolite specialization consistent with an earlier growth based study^21^,^22^. Only two metabolites, glutamate and unknown metabolite number 153 (m/z 330.1445 in positive mode, Supplementary Data 1), were depleted from the media by all seven isolates (Fig. 3, Supplementary Fig. 2). An additional seven metabolites were taken up by all six heterotrophic bacteria but not by *M. vaginatus*. Surprisingly, the overlap in utilization was small, and only 70 metabolites (19%) were consumed by at least four of the seven studied bacteria (Fig. 4b). No evidence of uptake by any of our isolates was found for 40% of the metabolites detected (Fig. 4b). Some metabolites were only released by a subset of isolates and not detected in any of the control media at a significant level. Individual isolates only used a small fraction of the available 372 metabolites: from 13−35% (Fig. 4c).

Interestingly, *M. vaginatus*, a photoautotroph, used the largest fraction of metabolites (Fig. 4c), which runs against the notion that cyanobacteria are not as competitive as heterotrophs due to a lack of effective uptake systems. Uptake of small organic compounds has been reported for cyanobacteria such as utilization of amino acids by *Prochlorococcus* sp.^46^ or utilization of a broad range of metabolites by *Synechococcus* sp. PCC 7002 revealed by exometabolomics^25^. Recycling of dissolved organic matter by cyanobacteria may thus be a significant factor shaping the composition and dynamics of these biocrust microbial communities. The necessarily small set of heterotrophic isolates tested are ‘specialists'^14^ and show a higher degree of specialization towards consumption of small subsets of available metabolites. We also find a large proportion of unused metabolites, which is consistent with this view of heterotroph specialization and exometabolite driven niche partitioning.

### Exometabolite dynamics in authentic biocrusts

To link these observations to the intact community and to determine the overlap of isolate metabolites with metabolites present in the natural soil environment we performed metabolite profiling of biocrust soil water using LC−MS. Since microbial metabolism in these desert biocrusts occurs during short pulsed wetting events, sometimes saturating soils^35^ we profiled changes in microbial metabolites at 3 min, 9 and 18 h following wetting of naturally desiccated biocrusts (Supplementary Fig. 3a). The abundance of metabolites in supplemented media was found to be roughly comparable to that of the biocrust soil water 3 min after wetting (peak areas within the same order of magnitude, Fig. 3, Supplementary Fig. 2) and the majority of metabolites detected in spent complex media data sets of biocrust isolates were also detected in biocrust soil water samples (Supplementary Fig. 3b,c, Supplementary Data 1). Overall, there was a decrease in the levels of 65 metabolites (*P*<0.05, Student's *t*-test) 18 h after wetting (Supplementary Fig. 3c) suggesting net uptake of these metabolites by biocrust microorganisms.

The diversity of metabolites detected in the crust soil water shortly after wetting is consistent with rapid release of metabolites via mechanosensitive channels during osmotic transitions^47^,^48^ or with cellular damage and metabolite leakage caused by re-wetting events^44^. Dihexoses, which are common intracellular compatible solutes^49^, appear to be released on wetting and then are depleted in biocrust soil water. These likely represent a significant resource in this environment and were consumed by most of the heterotopic isolates. Surprisingly, the primary producer, *M. vaginatus* is also found to take up the dihexose(s) while simultaneously releasing an array of other oligohexoses (described below). Glutamate, glutamine and citrulline are also depleted in both the biocrust soil water and in the complex media and are among the 76 metabolites that are taken up by at least two isolates but not released by any isolate (Fig. 3a,b and Supplementary Fig. 2). Potential competition for these metabolites involved in nitrogen metabolism is consistent with fixed nitrogen limitation in biocrusts and the inability of *M. vaginatus* to fix atmospheric nitrogen^37^,^43^. However, in what is an apparent paradox, all isolates are found to release other nitrogen-containing metabolites under these culture conditions (Fig. 3 and Supplementary Fig. 2), consistent with previous reports showing release of a large fraction of the fixed nitrogen^50^.

**Figure 5:**
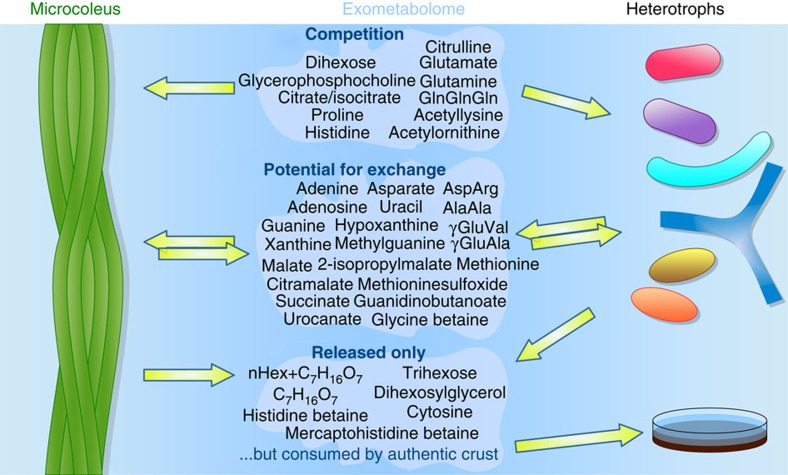
Uptake and release of putatively identified metabolites by individual isolates. The results show a potential for competition for a small groups of metabolites (only taken up by isolates and not released) as well as potential for cross feeding (metabolites released by at least one isolate and taken up by at least one other isolate). Some metabolites were released by isolates, not taken up by any other isolate but depleted by authentic biocrusts suggesting the presence of other microorganisms in the biocrust able to use these metabolites.

The observed substrate preferences provide a mechanism by which diverse soil bacteria avoid pure competition by specialization, yet in aggregate they metabolize complex carbon pools. Cross feeding is another known mechanism for stabilizing diversity^14^ and we observe 80 metabolites that may support cross feeding where uptake is observed for at least one isolate and release by at least one other isolate. Examples of these metabolites include the dipeptides aspartyl-arginine (the building block of cyanobacterial storage polymer cyanophycin) and gamma-glutamyl-valine released by *M. vaginatus* and consumed by some of the heterotrophic isolates. Other examples include common nucleosides or nucleobases (Figs 3,5 and Supplementary Fig. 2).

Finally, we found that a large fraction of metabolites (40%) are not used by any of the isolates (Fig. 4a,b and Supplementary Fig. 2) yet many of these were depleted with time in the intact biocrust. These include metabolites from *M. vaginatus* such as histidine betaine and mercaptohistidine betaine (possibly ergothioneine), which are common in cyanobacteria^41^,^45^,^51^ and a series of oligohexoses with a single C_7_H_16_O_7_ residue^45^. While these were not taken up by our isolates, the observed depletion in biocrust soil water points to the presence of other microorganisms that are able to use these compounds (Fig. 3 and Supplementary Fig. 2). This observation can guide isolation of microbes occupying other metabolite niches.

### Exometabolite analysis of isolates from mesophylic soils

To test if bacteria from a different environment exhibit similar substrate preferences, we selected six mesophilic soil isolates from OakRidge Field Research Center and performed a similar exometabolomic study. Metabolite extracts of the authentic soil samples were used as supplements to minimal M9 media. We detected 110 putative distinct metabolites, only 3 of which were used by all 6 isolates and 50 were not used by any. Similar to biocrust isolates, the mesophilic soil isolates used only subsets of metabolites with limited overlap (Supplementary Fig. 4).

It must be noted that the limited number of isolates used in these studies provide only a partial view into exometabolite niche partitioning and potential metabolic interactions among these microbes. Large studies across many environments would be required to support general conclusions and we anticipate that the addition of other isolates would increase overlap in metabolite usage. Given the diversity of soil metabolites, even small differences in metabolite utilization could play important roles in maintaining diversity. Especially when considering that specialization may extend to the utilization of unique combinations of substrates.

## Conclusion

Heterotrophic soil isolates were cultured to early stationary phase and exometabolomic experiments were used to assess the ability of individual isolates to uptake or release specific metabolites from relevant complex metabolite pools. This revealed narrow and largely non-overlapping substrate preferences for sympatric soil bacteria towards soil exometabolites that may indeed provide additional niche dimensions that help support diversity. Thus, the microbial community structure and carbon cycling may be linked to the diverse composition of extracellular metabolites including many yet unknown compounds. Environmental factors that affect biological diversity negatively may indirectly decrease the efficiency of biogeochemical cycling. Therefore, a better understanding of the reciprocity between metabolite and community composition has the potential to help integrate microbial community structures with global carbon cycles. Extension of these studies to include analysis of the dynamics of uptake and release and both the evolutionary and spatial context of substrate preferences hold great promise for improving our understanding of the processes governing chemical niche differentiation.

## Methods

### Chemicals

Water (Honeywell), acetonitrile (OmniSolv), and methanol (J.T.Baker) were LC−MS grade. All chemicals were obtained from Sigma.

### Bacterial strains, culture conditions, and biocrust samples

*M. vaginatus* PCC 9802 was isolated by F.G.-P. from the same area near Moab UT and crust types, and having been deposited into a public culture collection (Pasteur culture collection, PCC) it is referred to by its collection number. Six heterotrophic isolates (Table 1) were selected from our collection of biocrust isolates^40^ including both abundant and rare taxa. *M. vaginatus* was cultured in 50 ml of BG-11 media^52^ at room temperature under fluorescent lights (∼13 μmol photons × s^−1^ × m^−2^, 12 h light/12 h dark). Heterotrophic isolates were each cultured individually in 50 ml of a diluted 1:20 version of R2 media^53^ to obtain cell metabolite extracts to supplement minimal media in downstream exometabolomic experiments. Cell suspensions were distributed in 50 ml centrifuge tubes, centrifuged at 3,220*g* for 10 min, washed with PBS, and then each cell pellet was resuspendend in 1 ml of methanol. Methanol suspensions of *M. vaginatus* were sonicated for five cycles for 40 s using a sonication bath (Bransonic Ultrasonic bath, Model 2510R-DHT) at 40 kHz frequency and 24 °C temperature. Similar treatment was applied to heterotrophic strains. Methanol suspensions were centrifuged at 2,348*g*, supernatants were dried down using Savant SPD111V SpeedVac Concentrator and stored at −80 °C before being redissolved and supplemented to BG-11 media for exometabolomic experiments. Heterotrophic isolates were cultured to early stationary phase in the supplemented BG-11 media for exometabolomic analysis.

Biocrust samples (*n*=24) from Green Butte Site near Canyonlands National Park, Moab, UT, USA originated from the same sampling batch as in a previous study^35^. The biocrust samples in 6 cm^2^ Petri dishes were wetted with the addition of 10 ml of LC−MS grade water. The samples were held at room temperature and were illuminated with fluorescent lights (∼13 μmol photons × s^−1^ × m^−2^) for 12 h and then covered with aluminium foil to keep the samples in the dark. A total of 2 ml of soil water was sampled from each replicate. A total of eight replicates were sampled after 3 min (light), eight additional independent replicates were sampled after 9 h (light) and eight additional independent replicates were sampled after 18 h (dark). Soil water was replenished for the last eight replicates (dark) 9 h after wetting by adding additional 8 ml of LC−MS grade water to each replicate. Soil water samples were centrifuged at 2,348*g* for 5 min and 1.8 ml of the supernatant was used for metabolite extraction as described below.

For mesophilic soil isolates (Supplementary Fig. 4), five out of the six strains used in this study were isolated from groundwater collected from wells GW101, FW507 or FW104 situated within the OakRidge Field Research Center. Small subsamples of groundwater were streaked on R2A medium, and incubated under aerobic conditions at room temperature. Colonies developed within 72−96 h. *Pseudomonas migulae* strain N2C2 was isolated by streaking a small subsample of groundwater from well FW301 in anaerobic minimal fresh water medium^54^ with 10 mM sodium butyrate as the sole carbon source and electron donor and 10 mM sodium nitrate as the electron acceptor.

### Metabolite extraction and profiling

A total of 5 ml of spent and control media supernatants were frozen and lyophilized using Labconoco FreeZone 2.5 lyophilizer. Lypohilized samples were resuspended in 100 μl of methanol containing benzoic acid-2,3,4,5,6-D5 (with five deuterium substitutions) and alanyl−lysine as internal standards. The suspensions were centrifuged at 2,348*g* for 10 min. Supernatants were dried down under air flow, resuspended in 60 μl of methanol, filtered using 0.2 μm microcentrifuge PVDF filters (Merck Millipore) and analysed using LC−MS. A total of 1.8 ml of biocrust soil water samples were frozen and lyophilized. Lyophilized samples were resuspended in 100 μl of methanol with internal standards, filtered, and analysed by LC−MS.

LC−MS analysis was performed using normal phase liquid chromatography (Merck SeQuant ZIC-HILIC column, 150 × 1 mm, 3.5 μm, 100 Å) coupled to an Agilent 6520 ESI-Q-TOF as described previously^41^. The following LC conditions were used: solvent A, 5 mM ammonium acetate; solvent B, 90% acetonitrile with 5 mM ammonium acetate; timetable: 0 min, 100% B; 3 min, 100% B; 33 min, 0% B; 43 min, 0% B; 45 min, 100% B; 65 min, 100% B. Flow rate: 20 μl × min^−1^; injection volume: 1 μl. The acquisition was performed in fast polarity switching mode. Fragmentation (MS/MS) spectra were acquired using the same chromatography in single polarity modes using data-dependent precursor selection and collision energy of 10 V.

### Data analysis

Comparative analysis of control and spent media metabolite profiles was performed using the MathDAMP package^55^. Spectral features with signal intensities correlated in the retention time dimension were grouped as described previously to account for the multitude of possible ions per metabolite^25^. Mass spectral peaks within groups were inspected manually to assign ion types (for example, adducts, multimers and fragments) based on characteristic mass differences^56^. Chemical formulas were calculated for characteristic ions (usually [M+H]^+^, [M−H]^−^, or [M+NH_4_]^+^) based on accurate mass and relative isotopic abundance measurements using Agilent MassHunter software (version B.03.01). Metabolites were identified or putatively identified based on our previous studies^25^,^41^,^45^ and analysis of MS/MS data against spectral libraries Metlin^57^ and MassBank^58^. Peak areas of characteristic ions of metabolites were integrated using a ±20 mDa integration window for relative comparisons.

### Sequencing of the cyanobacteria attached community

To assess the structure of the community physically associated with the filamentous cyanobacteria, a method for removing sand from the filamentous cyanobacteria in the dry biocrust samples was employed. Briefly, eight 1 g biocrust samples were individually placed in 50 ml centrifuge tubes (Corning, Corning NY) and vibrated on the lowest setting of a Vortex Genie-2 (Scientific Industries, Bohemia NY) for ∼1 min and then centrifuged at 3,000 r.p.m. for 5 min (Eppendorph 5810R, Hamburg Germany) and this process was repeated approximately five times and they were then gently shaken until filament bundles were separated from the sand particles as shown in Supplementary Fig. 1a. Half of these samples were wetted with 500 μl sterile Millipore (Billerica, MA) water for 24 h (12 h sunlight) and then DNA from both the wet and dry biocrusts was isolated using the PowerSoil DNA Isolation Kit (MoBio, Carlsbad CA) using one tube per sample, following the protocol provided by the manufacturer. We used general prokaryotic primers targeting the 16S rDNA V4 hypervariable region: forward 5′- GTGCCAGCMGCCGCGGTAA -3′, reverse 5′- GGACTACHVGGGTWTCTAAT -3′. Each sample was amplified with a reverse primer customized with an adaptor, a primer pad, a specific Golay barcode and a linker based on Caporaso *et al*.^59^ protocol. The PCR was performed in triplicate for each sample and PCR products were pooled afterwards. The PCR was performed using the Takara ExTaq and reagents under these conditions: an initial phase of denaturation (4 min at 94 °C), followed by 24 cycles (denaturation at 94 °C for 20 s, annealing at 50 °C for 30 s, extension at 72 °C for 42 s), followed by a final extension phase (10 min at 72 °C). The PCR products were cleaned using 0.1% Seramag beads in buffer solution. After incubation on a magnetic plate for 2 min, the PCR supernatant was discarded. The beads were washed twice with 80% ethanol, the PCR products were eluted with 50 μl of 1x TE. Samples randomly picked were analysed with the Bioanalyzer (Agilent) to check their cleanliness. PCR products concentration was assessed by Qubit and pooled to achieve a final amount of 25 ng of DNA per sample in the library. The library DNA overall concentration was estimated using the KAPA SYBR FAST pPCR Kit following manufacturer's instructions (KapaBiosystems, Boston, MA, USA). The library was diluted to a concentration of 2 nM DNA then denatured by adding 0.2 M NaOH at room temperature for 5 min. A mix of 60% library, 40% PhiX was loaded on to the Miseq Illumina (500 cycles) cartridge for a final concentration of 20 pM DNA. The sequencing was performed on MiSeq using custom primers^59^, paired ends sequencing and default chemistry.

### Processing of iTag sequences

UPARSE method was used for sequence processing and operational taxonomic unit (OTU) clustering at 97% identity^60^ to process raw sequences (fastq_maxdiffs=3, fastq_trunclen=250, fastq_maxee=0.1). A set of 852 OTUs from a total of 239,859 filtered sequences from eight samples (four pre- and four post-wetup) were identified. For each OTU, a representative sequence was selected as in ref. 60. Taxonomic assignments were made with a Naïve Bayes Classifier, as implemented in Mothur^61^, using V4 region of the SILVA^62^ SEED sequences and their taxonomic identities as a training set.

### Comparison of isolates' and iTag 16S sequences

For each of the 6 isolates, V4 region from the full-length 16S gene was aligned to OTU representative sequences and the per cent identity, taxonomic classification and rank abundance of the best-matching OTU was noted. Supplementary Fig. 1 and Supplementary Table 1 show the rank-abundance curves for pre- and post-wetup samples with these OTUs marked.

## Additional information

**Accession codes:** The metabolomic data have been deposited in the MetaboLights database with accession code MTBLS211. The 16S rDNA sequence data have been deposited in the NCBI BioProject database with accession code PRJNA288975.

**How to cite this article:** Baran, R. *et al*. Exometabolite niche partitioning among sympatric soil bacteria. *Nat. Commun*. 6:8289 doi: 10.1038/ncomms9289 (2015).

## Supplementary Material

Supplementary Information Supplementary Figures 1-4 and Supplementary Table 1

Supplementary Data 1 Peak areas of characteristic ions of annotated metabolites in control media, spent media of biocrust isolates, and soil water from authentic biocrusts.

## Figures and Tables

**Figure 1:**
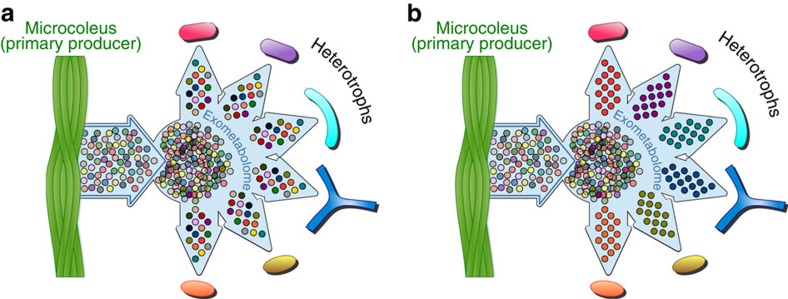
Two scenarios for substrate preferences in sympatric soil bacteria. (**a**) Metabolites have a weak effect in supporting diversity due to substrate generalism among heterotrophs. (**b**) Metabolite diversity contributes to niche differentiation and supports diversity due to strong substrate preferences among heterotrophs.

**Table 1:** Taxonomy of bacterial isolates used for exometabolomic analysis.

**ID**	**Phylum**	**Class**	**Order**	**Family**	**Genus**
	Cyanobacteria	Oscillatoriophycideae	Oscillatoriales	Microcoleus	*Microcoleus*
L1B56	Proteobacteria	Alphaproteobacteria	Rhizobiales	Bradyrhizobiaceae	*Bosea*
D1B20	Proteobacteria	Alphaproteobacteria	Rhizobiales	Methylobacteriaceae	*Methylobacterium*
L1B44	Actinobacteria	Actinobacteria	Frankiales	Geodermatophilaceae	*Modestobacter*
D1B45	Actinobacteria	Actinobacteria	Micrococcales	Micrococcaceae	*Arthrobacter*
L2B47	Firmicutes	Bacilli	Bacillales	Bacillaceae	*Bacillus* (1)
D1B51	Firmicutes	Bacilli	Bacillales	Bacillaceae	*Bacillus* (2)

*M. vaginatus* PCC 9802 and the six heterotrophic isolated from Biocrusts near Moab UT^40^.
