# Supplementary material for: Exometabolite niche partitioning among sympatric soil bacteria
Source: Nat Commun. 2015 Sep 22;6:8289. doi: 10.1038/ncomms9289 (PMC4595634; doi:10.1038/ncomms9289)
Supplement: Supplementary Information — Supplementary Figures 1-4 and Supplementary Table 1 [file ncomms9289-s1.pdf]

a

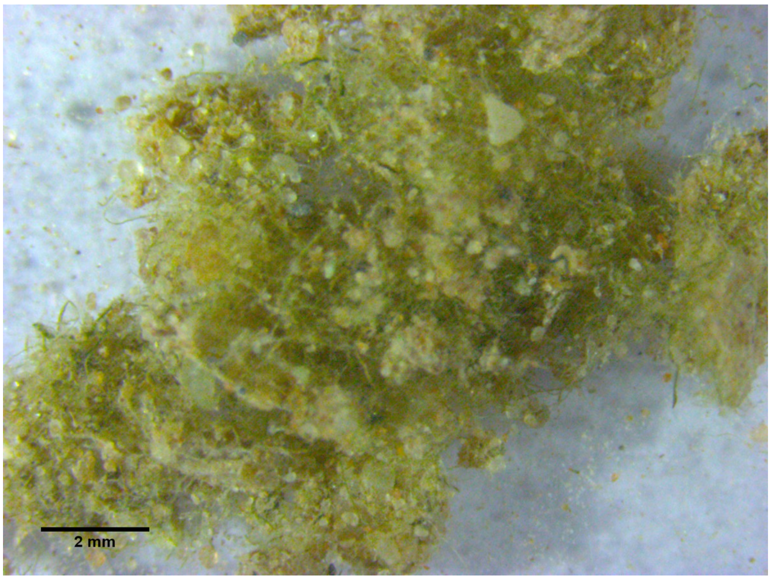

b

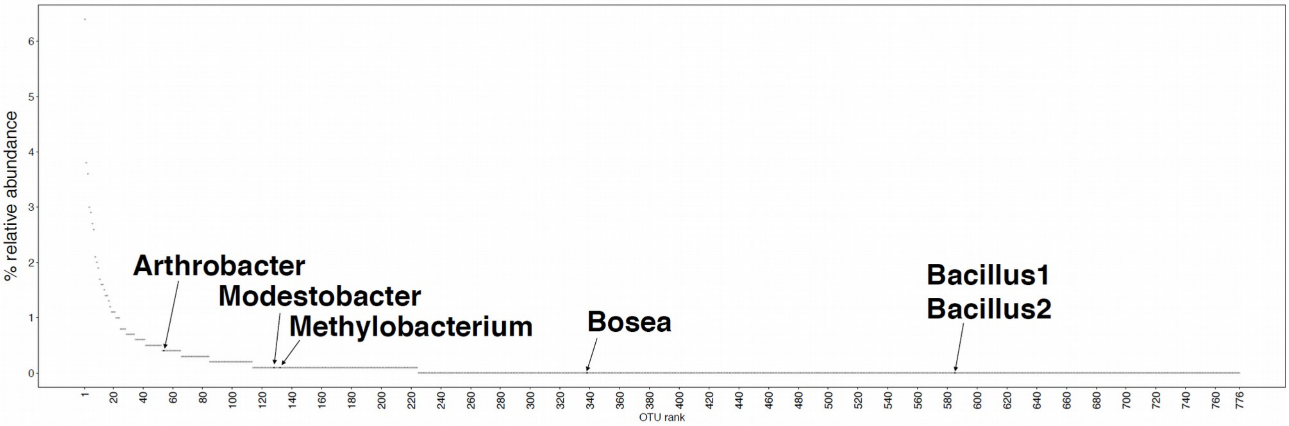

c

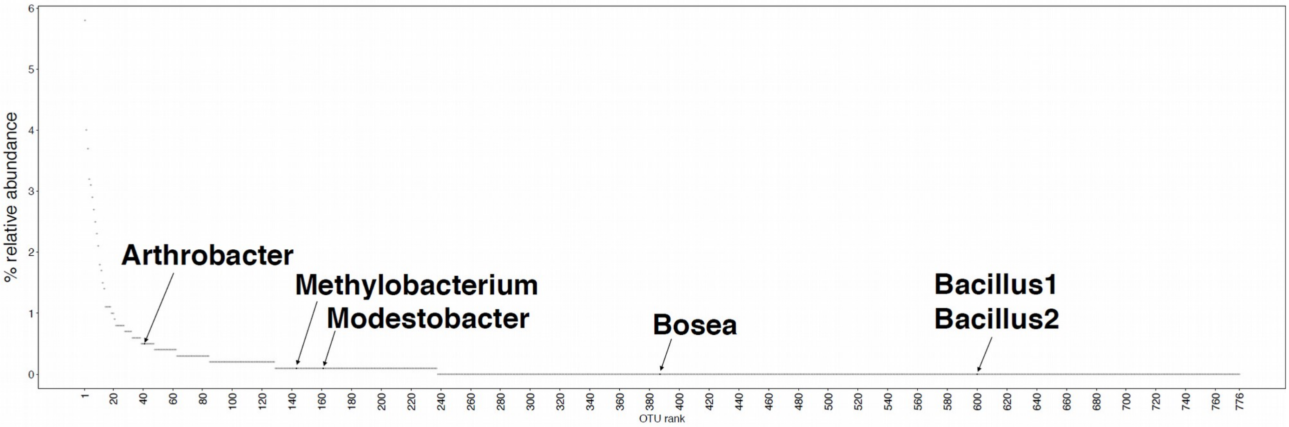

Supplementary Figure 1 | Biocrust microbial communities. (**a**) Photograph of an example of the cyanobacteria associated community [Image taken using Leica (Wetzlar, Germany) M165 FC dissection microscope complete with a Planapo 1.0x objective, DFC400 detector and KL 1500 LED light source]. Rank abundance curves for the community pre- (**b**) and post- (**c**) wetup. For each isolate, the closest OTU (with the highest percent identity) is marked.

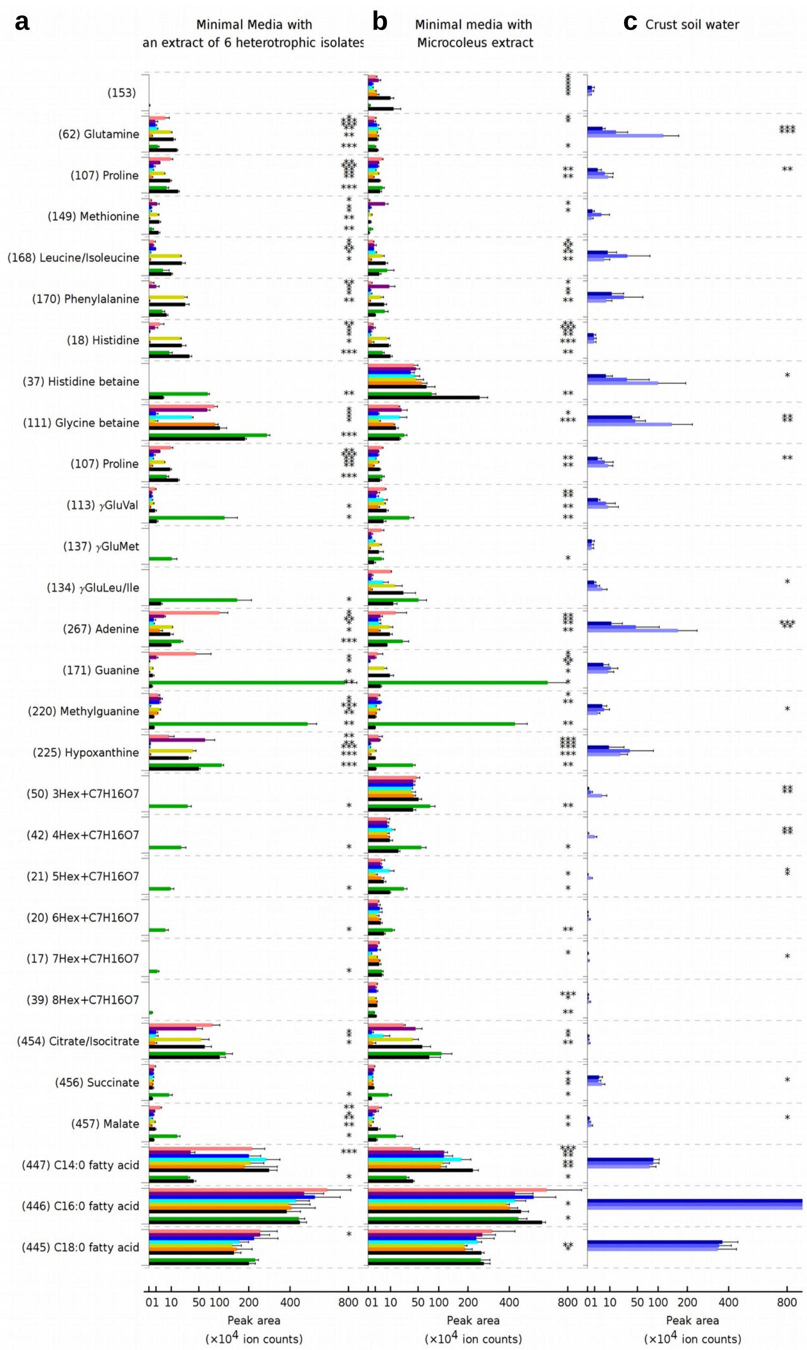

Supplementary Figure 2 | Uptake and release of selected metabolites by biocrust isolates (a, b) and authentic biocrust (c). Individual isolates were cultured in minimal media supplemented with a pooled metabolite extract of six heterotrophic isolates (**a**, Table 1) or metabolite extract of *Microcoleus vaginatus* PCC 9802 (**b**). (**a**, **b**) Levels of metabolites in spent media (colored bars) were compared to control media (no cells, black bars) to detect uptake or release of corresponding metabolites (n = 3). A separate set of control media (black bars next to green bars) were used for Microcoleus spent media as Microcoleus was cultured for a significantly longer time than the heterotrophs. (**c**) Levels of selected metabolites in crust soil water extracts (Supplementary Fig. 3) are also shown (n = 8). Error bars correspond to standard deviations. Stars correspond to Student's *t*-tests between spent media (colored bars) and the corresponding control media (black bars; * – $p < 0.05$; ** – $p < 0.01$; *** – $p < 0.001$). Figure 3 in the main text shows these comparisons for additional metabolites.

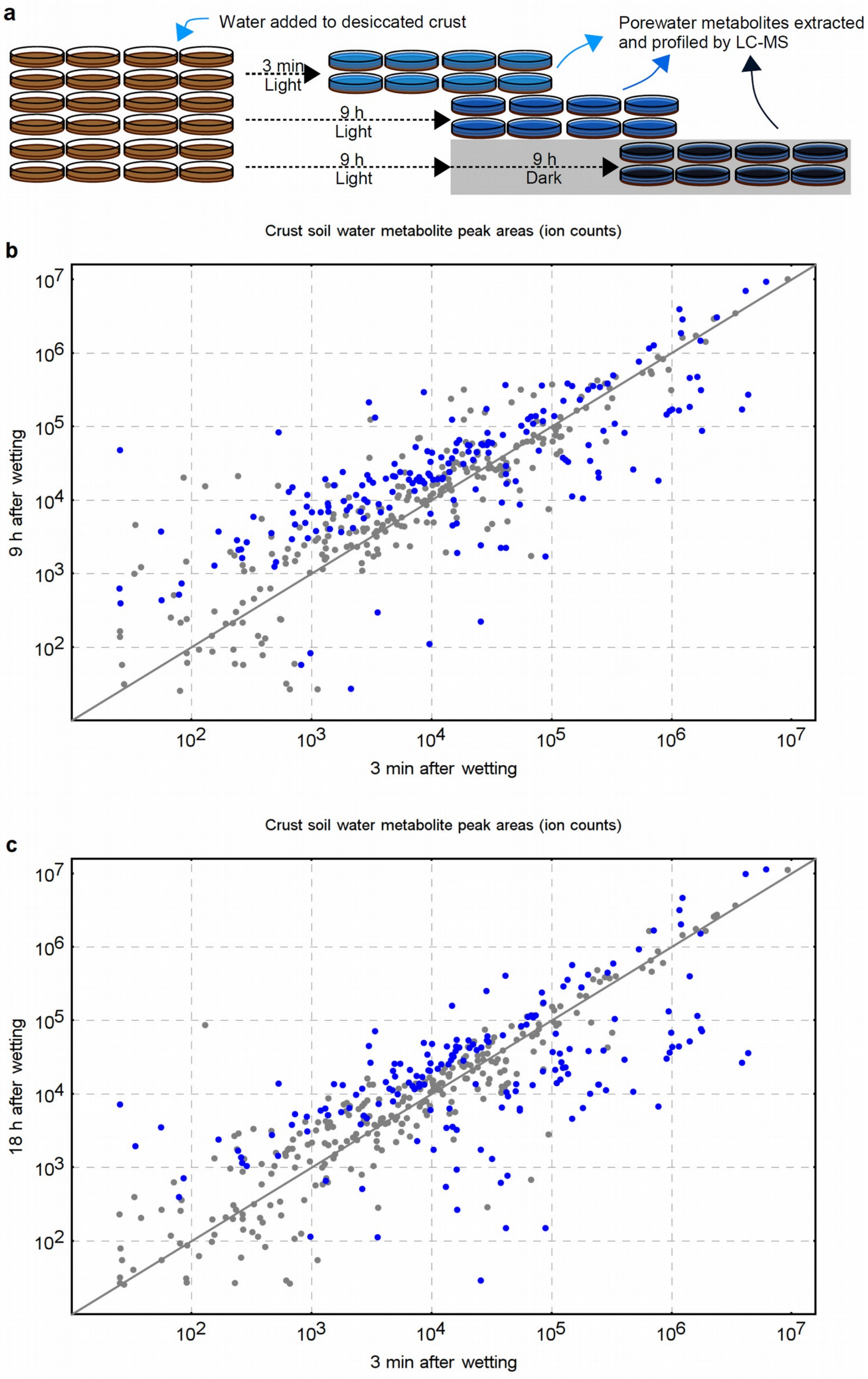

Supplementary Figure 3 | Release and uptake of metabolites by biocrust following wetting. (**a**) Water was added to desiccated biocrusts; and to simulate initial wetting, a light cycle, and a light-dark cycle, soil water metabolites were extracted 3 min, 9 h or 18 h respectively following wet-up. (**b**) Levels of metabolites shown in blue were significantly different ($p < 0.05$, Student's *t*-test) between 3 min soil water extracts and 18 h extracts showing initial release of metabolites into the soil water followed by uptake of a subset of the metabolites by biocrusts (n = 8).

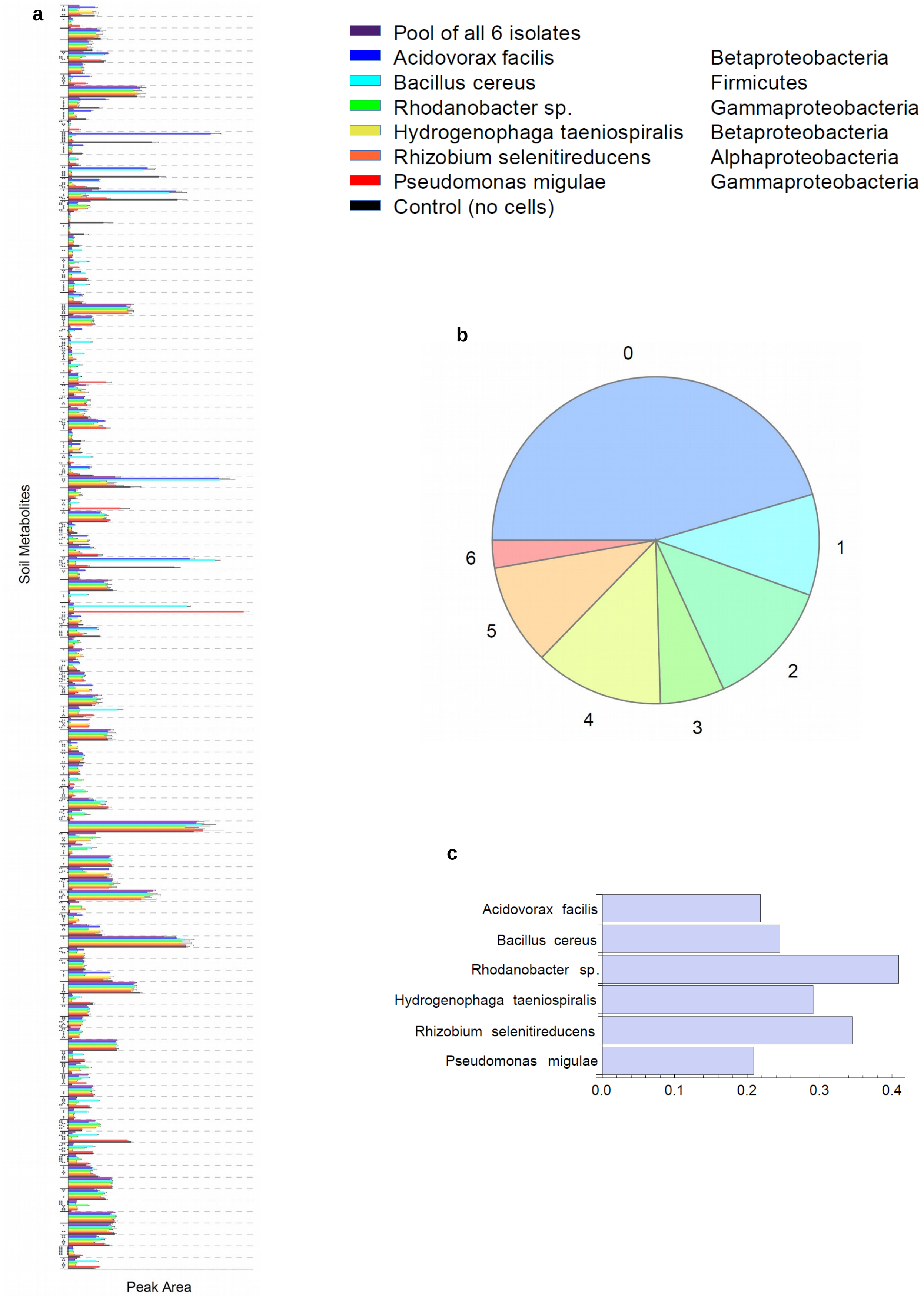

Supplementary Figure 4 | Uptake and release of metabolites by mesophilic soil isolates (a). Similar to biocrust isolates (Fig. 4), mesophilic soil isolates utilize only subsets of metabolites with limited overlap (**b**, **c**).

Supplementary Table 1

	Most similar OTU	Percent Identity (V4)	Dry: % relative abundance within non-Cyanos	Dry: Rank	Wet: % relative abundance within non-Cyanos	Wet: Rank	OTU Taxonomic Classification
**Methylobacterium**	**OTU_118**	98%	0.1	132/776	0.1	143/776	k__Bacteria; p__Proteobacteria; c__Alphaproteobacteria; o__Rhizobiales; f__Methylobacteriaceae; g__; s__
**Arthrobacter**	**OTU_44**	96%	0.4	54/776	0.5	41/776	k__Bacteria; p__Actinobacteria; c__Actinobacteria; o__Actinomycetales; f__Micrococcaceae; g__; s__
**Bacillus 1**	**OTU_726**	95%	0.0064	585/776	0.0001	600/776	k__Bacteria; p__Firmicutes; c__Bacilli; o__Bacillales; f__Bacillaceae; g__Bacillus; s__
**Modestobacter**	**OTU_434**	97%	0.1	128/776	0.1	161/776	k__Bacteria; p__Actinobacteria; c__Actinobacteria; o__Actinomycetales; f__Geodermatophilaceae; g__Modestobacter; s__
**Bosea**	**OTU_371**	93%	0.022	338/776	0.0001	387/776	k__Bacteria; p__Proteobacteria; c__Alphaproteobacteria; o__Rhizobiales; f__Bradyrhizobiaceae; g__; s__
**Bacillus 2**	**OTU_726**	94%	0.0064	585/776	0.0001	600/776	k__Bacteria; p__Firmicutes; c__Bacilli; o__Bacillales; f__Bacillaceae; g__Bacillus; s__
